# Using structural motif descriptors for sequence-based binding site prediction

**DOI:** 10.1186/1471-2105-8-S4-S5

**Published:** 2007-05-22

**Authors:** Andreas Henschel, Christof Winter, Wan Kyu Kim, Michael Schroeder

**Affiliations:** 1 Biotechnological Center, TU Dresden, Tatzberg 47-51, 01307 Dresden, Germany; 2 Institute for Cellular and Molecular Biology, University of Texas at Austin, Austin, TX 78712, USA

## Abstract

**Background:**

Many protein sequences are still poorly annotated. Functional characterization of a protein is often improved by the identification of its interaction partners. Here, we aim to predict protein-protein interactions (PPI) and protein-ligand interactions (PLI) on sequence level using 3D information. To this end, we use machine learning to compile sequential segments that constitute structural features of an interaction site into one profile Hidden Markov Model descriptor. The resulting collection of descriptors can be used to screen sequence databases in order to predict functional sites.

**Results:**

We generate descriptors for 740 classified types of protein-protein binding sites and for more than 3,000 protein-ligand binding sites. Cross validation reveals that two thirds of the PPI descriptors are sufficiently conserved and significant enough to be used for binding site recognition. We further validate 230 PPIs that were extracted from the literature, where we additionally identify the interface residues. Finally we test ligand-binding descriptors for the case of ATP. From sequences with Swiss-Prot annotation "ATP-binding", we achieve a recall of 25% with a precision of 89%, whereas Prosite's P-loop motif recognizes an equal amount of hits at the expense of a much higher number of false positives (precision: 57%). Our method yields 771 hits with a precision of 96% that were not previously picked up by any Prosite-pattern.

**Conclusion:**

The automatically generated descriptors are a useful complement to known Prosite/InterPro motifs. They serve to predict protein-protein as well as protein-ligand interactions along with their binding site residues for proteins where merely sequence information is available.

## Background

Exhaustive knowledge about protein interactions is a prerequisite to understanding the molecular machinery of the cell. While comprehensive protein sequence databases are available, the number of known PPIs is still small. In addition, experimentally proven PPIs often do not reveal the binding sites involved. The implications of the discovery of binding sites are manifold: the discovery of patterns in amino acid arrangements is of general importance in the study of protein-protein interactions. Furthermore, docking algorithms benefit greatly from the correct prediction of binding sites. Finally, interaction prediction is the key to mapping global interaction networks and signalling pathways, and may help elucidate the functions of individual proteins. Complementary to experimental techniques are computational approaches that analyze and predict protein-protein interactions. Sequence-based methods include gene context conservation [1], synthetic lethality [2], phylogenetic profiling [3,4] or co-evolution of gene expression [5]. Various databases of binding sites and interfaces between proteins and their domains exist [6-8]. An extensive list of prediction methods can be found in [9].

**Figure 1:**
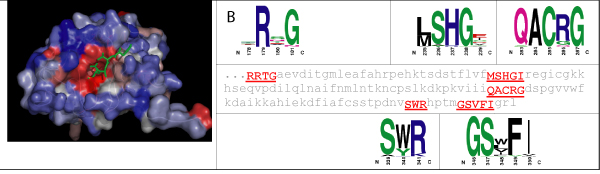
**Constructing a set of sequence profiles to represent a conserved structural feature**. Caspase's active site is highly conserved (1ICE, conservation levels are calculated using the von-Neumann entropy and displayed in a color gradient from blue (variable) to red (conserved)). Conserved residues in close vicinity of the tetrapeptide inhibitor largely define the catalytic site environment. Caspase residues within 5 Å of the inhibitor are underlined. Segments are patched and those with low conservation are discarded to avoid insignificant hits. We add the amino acid distribution from HSSP data for each site of the remaining segments. It is thus possible to construct HMMs and visualize the profiles as sequence logos [40].

Functional characterization of novel genes and their proteins remains an important and challenging task. It often is improved by the identification of novel interaction partners. It has been observed that structural and functional features of proteins like catalytic sites are often well conserved [10]. In contrast, the rest of the surface is often more variable (see Figure 1A), which impedes sequence similarity searches for functionally equivalent or similar proteins. Descriptors previously used for conserved domains and interface motifs include regular expressions, weight matrices, and profile Hidden Markov Models (HMMs). These descriptors involve either sequentially consecutive stretches [11-13] or full length domains [14]. In particular, HMMs were successfully employed in many sequence similarity search tools [14-16].

As pointed out by Bailey and Gribskov [17], the signal-to-noise ratio in homology searches can be improved by using sets of motifs that characterize a family. In this study, we aim to create descriptors for all relevant sequence parts of structurally known protein-protein and protein-ligand binding sites. These binding sites are often well-conserved [18], but their segmented nature on sequence level has to be taken into account for sequence similarity searches (Figure 1B). In accordance with previous work [19,20], we define an interface between two proteins to consist of two faces. Similar faces can be clustered geometrically into face types, and similar interfaces can be clustered into interface types [20].

Many approaches for interaction prediction and function annotation require structural knowledge about the protein of interest [21-23]. By waiving this requirement, interaction prediction is applicable to a much wider range of sequences but becomes a substantially harder problem. It has been addressed previously (see e.g. [24,25]). Most notably, Li and Li [13] discover stable and significant interface motifs and represent them with regular expressions, while Espadaler *et al. *[12] prove the usefulness of HMMs for this endeavor. Both approaches use single structural templates as seeds for generalization with further sequences, coming from either similarity search or random generation. However, several structures for a particular kind of domain-domain interaction are available, each providing new insights into the sequential variability of the actual interacting residues. Novel to our method is the incorporation of as many structures as possible for each binding site descriptor. The benefits are intriguing as protein-protein interactions from complex structures are considered to be the most reliable source of interaction data.

## Face descriptors

We compiled a library that comprises profile HMM descriptors for 740 protein-protein and 3000 protein-ligand binding sites in the Protein Data Bank PDB [26]. Each descriptor describes one face. These descriptors, totalling more than 3,740, characterize an interaction/ligand binding site on sequence level. Hence, given a query sequence of interest, it is possible to compare it to each interface descriptor, thus identifying binding sites to possible interaction partners including ligands. Gene Ontology (GO) [27] annotations are linked to each descriptor from the original PDB entries that were used for its construction. The complete list of profile HMM descriptors is directly usable with the HMMer package [28] and is freely available for academics upon request.

### PPI descriptor construction

Based on the family level of the Structural Classification of Proteins, SCOP [29], we can extract and classify all domain-domain interactions found in the PDB. This classification is available in the SCOPPI database [30]. As pointed out by Kim *et al*., even homologous domain pairs can associate in geometrically different ways by employing different sets of residues to form interfaces [19,20]. Consequently, the corresponding interface profiles would differ substantially and combining the information about interacting residues to a profile would be meaningless. However, often a number of domain-domain interactions expose striking similarities and it is desirable to collect all instances of one interface type for the calculation of the respective interface profile. We therefore compose descriptors for all interface types in SCOPPI by combining all instances of that interface type. When data for interface types is sparse, we utilize sequence data provided by HSSP [31]. Often several sequentially remote segments contribute to a binding site. To accommodate for this phenomenon, we adopt the multiple-motif approach from PRINTS [32], MAST [17] and Meta-Meme [33] to represent binding sites as a collection of small HMMs for one local binding motif. Thus we describe only the important sequence parts that form a structural feature. To represent the full sequence space of a whole family with a weight matrix or a profile-HMM, a large number of sequences is required, in particular for families of strong sequence variability. However, considerably fewer sequences are needed for short, conserved motifs.

### PLI descriptor construction

As large protein complexes are often difficult to crystallize, knowledge about protein-protein interactions can be drawn from the more abundant protein-peptide interactions. The descriptor construction method can be extended to these cases in particular and to PLI in general. We construct HMM profiles for faces that bind to small molecules and peptides. To this end we scanned PDB for most frequently occuring co-crystallized ligands. We identify the residues surrounding the ligand incl. possible cofactors. A profile is solely built from one structural template and aligned sequences utilizing HSSP [31].

## Evaluation

**Figure 2:**
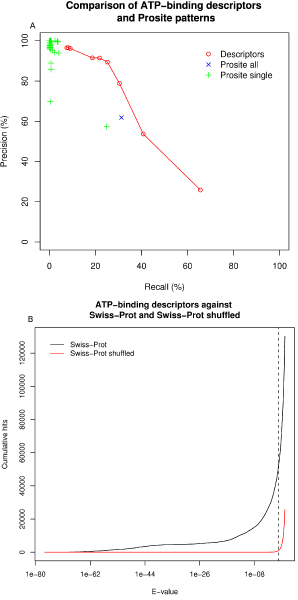
**Assessing accuracy and significance of ATP-binding descriptors**. **A**. Precision-recall curve for ATP-binding descriptors derived from protein structures with bound ATP or ADP tested against Swiss-Prot, shown as curve with red circles. Each circled point corresponds to a different E-value cutoff. The Prosite patterns for "ATP-binding" and "ADP-binding" are included as well (green crosses). Overall, Prosite achieves a recall of 31% with a precision of 62% (blue cross). For all E-values, our method performs better than Prosite. **B**. Distribution of E-values for the ATP-binding descriptors. To assess the significance of hits, the descriptors were tested both against Swiss-Prot (black line) and a shuffled Swiss-Prot version (red line). The cumulative number of hits below a certain E-value threshold is shown. The inlet shows a magnification of the lower right corner. Below an E-value of 1 (dotted vertical line), ~53,000 hits are found in Swiss-Prot whereas only ~1,200 hits are found in the shuffled Swiss-Prot.

In order to assess the significance of HMM scores, a number of comparisons to shuffled databases were conducted. Figure 2B demonstrates the expressive power of low E-values achieved with interface type HMMs against Swiss-Prot vs. shuffled Swiss-Prot. Random hits generally only occur with E-values above 1, while hits in Swiss-Prot below 1 can be considered significant.

### Assessing the performance of PLI descriptors

#### Benchmarking HMMer E-values

Expectation values provided by the HMMer software are a means for assessing the significance of HMM hits. As demonstrated by Li *et al*. [13], the statistical evaluation to randomness can be used to establish a Z-score to distinguish significant from random hits. Here, we use comparisons to shuffled databases to gain further information about the significance: by calculating the ratio of best E-values of hits from shuffled and not shuffled sequence databases. For database shuffling, we generated a random permutation for every single sequence in the database. In the sequel, we demonstrate the use of shuffled databases for one particular ligand – adenosine triphosphate (ATP).

#### Case study: ATP-binding sites

We evaluate ligand-binding descriptors for the case of ATP. The descriptors for ADP-binding and ATP-binding were run against 205,000 Swiss-Prot-annotated sequences and could extract 10,349 hits whereof 9,255 were true positives and 1,094 false positives. Given that 36,774 sequences were annotated as ATP-binding, this amounts to 25.2% recall and a precision of 89.4%. Prosite's P-loop motif recognizes 24.87% but in contrast produces 6792 false positives (precision: 57.4%). Figure 4 shows an overview of these numbers.

The precision-recall curve (Figure 2A) for our descriptors was generated from true/false positive rates at different E-value thresholds (Table 1). Scores for regular expressions from Prosite are generally below this curve. Prosite improves on this by adding highly specific full length sequence profiles with high precision but very low recall.

Some descriptors exhibited great similarity to the P-loop motif, e.g. the descriptor derived from PDB entry 2BEK, a chromosome partitioning ATPase (Figure 5): the central binding motif conserves Glycine and Lysine that build hydrogen bonds to ATP's phosphate tail. The Prosite pattern only disagrees on its first position (requires Alanine or Glycine, mostly Glutamine found) and does not give any specification to the following four residues. These however, are found to be well conserved in a wide variety of ATP-binding homologs. We thus have automatically developed a variation of the P-loop motif which is more precise for a range of orthologous chromosome partitioning ATPases that are picked by HSSP in other species.

**Figure 3:**
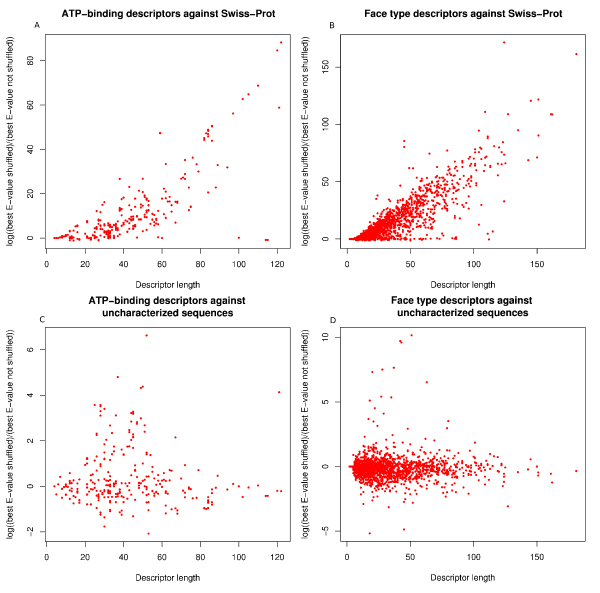
**Correlation of length and quality of HMM descriptors**. ATP-binding descriptors as well as face type descriptors for protein-protein interactions were run against original and shuffled versions of Swiss-Prot and uncharacterized NCBI sequences. We define the length of a profile Hidden Markov Model descriptor as its number of states. Quality is measured as difference between log E-values of best hit against original sequences and shuffled sequences. For Swiss-Prot, longer descriptors have better quality and therefore produce more significant hits. For uncharacterized sequences, this does not hold. One explanation could be that these sequences are depleted of significant matches by similarity searches.

**Figure 4:**
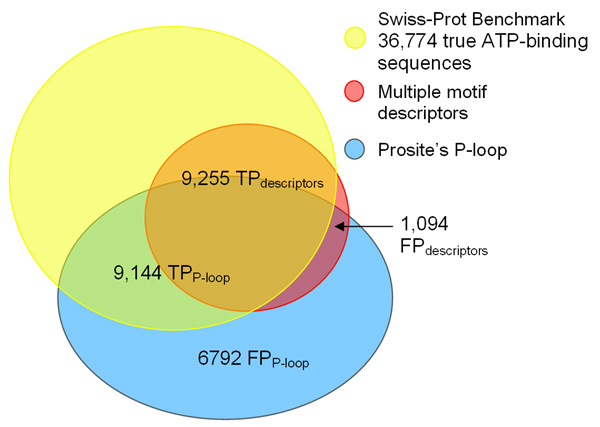
**Validation of PLI-descriptors**. Prediction of sequences annotated with the Swiss-Prot keyword „ATP-binding“ (or ATP/ADP as part of the catalytic activity) using Prosite patterns and multiple motif descriptors with hits below E-value 1: while both methods detect ~9.000 of all proteins annotated with this term by Swiss-Prot, Prosite provides almost sevenfold more false positives.

**Figure 5:**
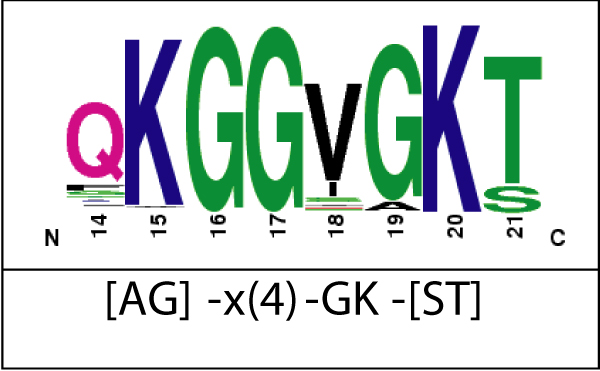
**ATP binding motifs**. The ATP-binding descriptor derived from PDB entry 2BEK and HSSP is compared to the the P-loop pattern (below). A single conflict occurs in the first position, and more specific detail is given about the second to the fifth residue. The descriptor therefore correctly detects other chromosome partitioning ATPases.

Figures 3A–D illustrate the use of E-value ratios of unshuffled vs shuffled sequence databases. In case of the ATP-binding descriptors searched against Swiss-Prot, the comparison of the two E-value distributions (shuffled/not shuffled) allows the identification of a significance threshold (Figure 2B).

Note that small motifs like e.g. the poly-proline (PxxP) motif occur frequently in sequence where only 5% are functional. Nevertheless, hits of small motifs are helpful to identify candidate sets that should undergo manual postprocessing.

### Assessing the performance of PPI descriptors

#### Cross validation with structure data sets

The initial data set comprised 740 interface descriptors of protein-protein interactions, each having at least three non-redundant instances (not more than 98% sequence identity). In order to check the ability of face descriptors to recognize faces from structures in the PDB, we performed 2-, 4- and 8-fold cross validations on interface types with at least 8 non-redundant instances. This yields a set of 45 interface types, i.e. 90 face types. This set was run against domains from PDB that are classified by SCOP. For 61 face types (67%), a reasonable recall of 70%–100% was achieved (additional file 1). Face types with low recall often have small interfaces with short, dispersed segments producing insignificant hits or have low face conservation. Another source of errors is misalignments of sequences of an interface type. In five cases, the recall could be improved by adding homologous sequences from HSSP [31]. The recall for interaction prediction by requiring both face types to be present is upper bound by the minimal recall of both faces. Hence, the average recall for interface detection dropped to 39%. This problem is most eminent for predicting interactions of promiscuous faces. Using the Structural Classification of Protein-Protein Interactions (SCOPPI [33]), it is then still possible to provide candidate interaction partners. In particular, for dedicated faces, i.e. those with just one opposite binding face, recognition of one face type suffices.

#### Literature protein-protein interaction benchmark

To investigate how well our method is suited to detect protein-protein interactions, we benchmark it against a set of high quality literature-curated interactions. To this end, we use a subset of NetPro, an expert curated and annotated database containing ~15,000 protein-protein interactions [34]. These were extracted from PubMed abstracts by a semi-automated method and then cross-checked by human experts.

Using 740 multiple motif descriptor pairs, we search the NetPro benchmark set for matches where a descriptor pair matches two interacting proteins. If we maximally relax our E-value cutoff criterion, we are able to predict ~80% of the literature interactions. At a stricter E-value of 0.001 we still validate 230 interactions (See details in Table 2).

### Using the descriptors to annotate uncharacterized sequences

We obtained a corpus of 32,000 uncharacterized proteins from the NCBI's non-redundant protein sequence database. Face descriptors were run against these sequences and a shuffled version. The result is shown in Figure 3D. A number of hits can be identified in the upper left part that have significant difference between log E-values of best hit against original sequences and against best hit of shuffled sequences. One example is the uncharacterized Fe-S protein from *Yersinia bercovieri *(NCBI ZP_00820831).

The face descriptor constructed from *E. coli *Complex II 2Fe-2S ferredoxin domain (which binds to the N-terminal domain of succinate dehydrogenase/fumarate reductase flavoprotein) matches this protein with an E-value of 4.4e-7. In contrast, the same descriptor achieves only 0.057 as best E-value when run against the shuffled version of the uncharacterized sequence database.

gi|77956751|ref|ZP_00820831.1|: score 14.5, E = 4.4e-07

ferredoxin_binding_site *->yRrSCReavCGSdviR.vD<-*

SCR ++CGS+ i vD

ZP_00820831 --YSCRAGICGSCRITlVD

This suggests that uncharacterized Fe-S protein could be part of the Complex II of *Yersinia bercovieri *interacting with succinate dehydrogenase flavoprotein subunit. The latter was found to be present in the available Yersinia pestis genome via sequence similarity search.

In order to estimate the quality of descriptors, E-value ratios for all descriptors in shuffled and original databases were analyzed in dependence of descriptor length (Figure 3). Not surprisingly, most descriptors perform well against SwissProt, in particular long descriptors. Significant hits are rarer in uncharacterized sequences, which had less or no influence in the generation of descriptors (Figure 3C,D). Interestingly, the best results were achieved by short and medium size descriptors. This suggests that long descriptors are less likely to discover binding behaviour in unknown sequences.

## Applications

The match of a descriptor pair allows identification of the putative residues responsible for the interaction. This information can be used to guide site-directed mutagenesis experiments that aim at disrupting the interaction.

The proposed method for binding site prediction can be applied by itself or in combination with other methods. A common technique for protein interaction prediction is by identifying interacting homologues. This concept of so-called interologues was first noted by Walhout et al. [35] and was applied in PPI prediction in e.g. [36,37]. The usage of descriptors can serve here as a refinement: the assumption that the residues responsible for the interaction are present can be easily confirmed by descriptor matches of the according families. Since a match provides residue correspondences to the original structures used to create the descriptor, the match alignment serves as an initial setup for homology modeling of the interface region.

## Limitations

For some interface types only few or no structures are available. This implies that protein-protein interaction descriptors are inaccurately or not at all represented by the descriptor library. On the other side, a pair of homologous sequences from HSSP does not necessarily preserve the interaction of the structural template and thus does not belong to a certain interface type. These sequences "contaminate" the alignments of interface descriptors. It is therefore essential to assure that recruited sequence pairs not only maintain an interaction but also agree in interface type, i.e. have similar sets of interface residues.

A related problem is that of interaction specificity. An interface descriptor with *N *and *M *hits for each face, respectively, induces *N *× *M *candidate interaction pairs. Aytuna et al. [38] argue analogously for structural face descriptor pairs and Deane et al. [37] verify interactions between pairs of yeast proteins by known paralogous interactions. Although the latter report only 1% false positives, results should be – as with any computational method – ideally supported by further evidence from experimental results.

Our technique to construct interface descriptors is inadequate for short, strongly dispersed or highly variable interfaces (e.g. loops in immunoglobulins). However, it is possible to create a descriptor with our method that spans over the surrounding secondary structures, which are often well conserved.

## Conclusion

We provide a library of Hidden Markov Model based descriptors that capture important structural features such as protein interfaces, ligand binding sites and active sites of enzymes. The implications for predicting binding sites and binding partners of proteins are many-fold. It provides insights into the biological processes the matched protein might be involved in. Furthermore the method can pinpoint interacting residues. It thus bears the potential for functional annotation and for assisting in discovering new drug targets.

Cross validation with the available structural data for a number of interface types reveals that two thirds of the face descriptors have a recall between 70% and 100%. Interaction prediction by recognizing both faces is intrinsically harder than just one-sided binding site detection. The cross validation results reflect this fact, as the recall for predicted interactions drops to 39%.

To demonstrate the biological significance of our descriptors, we compare the descriptors to NetPro, a PPI database with literature evidence. This way we could validate the predicted interactions and moreover provide insights about the critical interacting residues.

We created a benchmark for ATP-binding site detection. From a database of Swiss-Prot annotated sequences, our descriptors successfully recognized 30% while producing much less false positives than Prosite's regular expression for the P-loop motif.

Finally, an example for a significant hit for a binding site in an uncharacterized protein from *Yersinia bercovieri *is presented, which suggests a possible function as Complex II (succinate dehydrogenase) subunit for this protein.

## Methods

**Figure 6:**
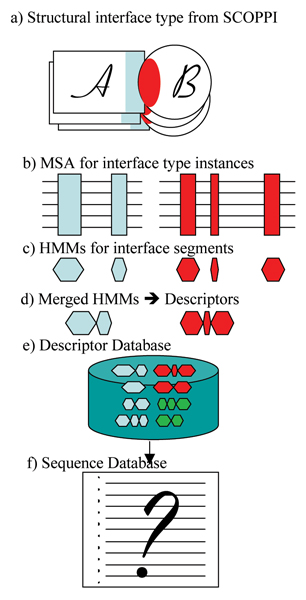
**Work flow**. a) All instances for interactions between family A and family B with identical geometric interface classification are retrieved from SCOPPI. Interface residues are indicated in the accompanying multiple sequence alignments. b) Interface columns are defined by columns with more than 50% interface residues. Interface segments are defined by including interface flanking columns. c) HMMs are constructed for each interface segment using HMMer's hmmbuild. d) HMMs are merged by insert states with high self-loop probabilities as to model the non-interacting linker region e) The collection of all merged HMMs constitutes the descriptor library. f) Sequence searches against Swiss-Prot and uncharacterized sequences with all descriptors were done using HMMer's hmmsearch

The workflow of our method is illustrated in Figure 6. The method uses protein structural data that describes a binding site to generate Hidden Markov Models. These are then used to search Swiss-Prot and uncharacterized sequences.

### Protein-protein interaction (PPI) descriptors

**Figure 7:**
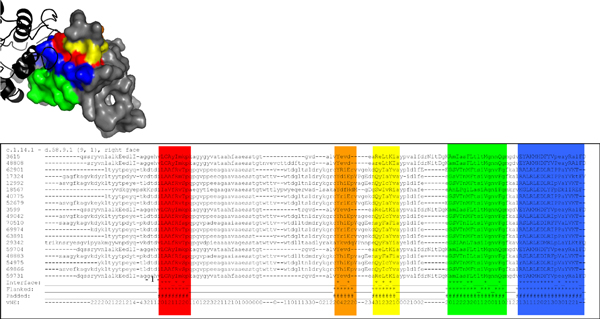
**A sample alignment for RuBisCo N-terminal domain**. Interacting segments are highlighted in corresponding colors in the alignment as well as in the structure. Interface segment definition is illustrated by the three lines below the alignment. Finally, the 10 fold von Neumann entropy is printed.

For PPI descriptors, data is taken from the SCOPPI database [30]. SCOPPI is a collection of domain interactions and their interfaces of proteins in the Protein Data Bank (PDB). An interface in SCOPPI consists of two faces. Similar faces are clustered into face types, and similar interfaces are clustered into interface types [20]. For this study, we use a non-redundant set of domain sequences for every interface type at 98% sequence identity. SCOPPI provides a multiple sequence alignment (MSA) constructed with MUSCLE [39] for domains of the same SCOP [29] family. Figure 7 shows such an alignment for one face type. Interacting residues are depicted in uppercase. Columns of the MSA are marked as interface columns if 50% of the sequences contain an interacting residue at that position. Interface columns are extended by first adding adjacent columns (flanking) and then filling gaps of length 1 (padding). The results are segments of continuous interface columns. For every segment, a Hidden Markov Model (HMM) is generated with the HMMer software [28]. Segment HMMs are merged into one HMM by linking them with insert states. The resulting HMM serves as face type descriptor for a PPI. The linking of HMMs is adopted from Meta-Meme [32], with the difference that insert and delete states remain unchanged in our approach. The probability for a self loop in the segment linking insert states is set to *l*/(*l*+1), where *l *is the average length of the linker region between two interacting segments. Finally, the merged HMMs are calibrated using HMMer's calibration with 5000 random sequences. The resulting HMMs are directly usable with the HMMer package [28] which provides sound E-value calculation without assuming segment scores to occur independently.

Despite the fact that homodimers account for a large set of interface types, we omit them because it is often unclear whether a homodimer interface is genuine or an artifact from crystallization.

### Protein-ligand interaction (PLI) descriptors

Due to the current lack of a comprehensive classification for geometrical associations of protein-ligand interactions, we generate PLI descriptors from single structural templates:

0. For each structure in the PDB containing a protein and a ligand:

1. Select a ligand

2. Iteratively expand the selection to include surrounding cofactors

3. Identify the residues surrounding the ligand selection within 4.5 Angstrom

4. If this yields at least three well conserved residues:

5. Include direct well conserved sequence neighbours

6. Include residues that are between selected sequence neighbors

7. Add sequences identified as structural homologous by HSSP

8. Generate HMMs for each segment

9. Combine HMMs into one descriptor connected by inserting states that reflect the linker regions between segments

10. Add descriptors (one for each ligand) to library

11. The library can now be used to predict ligand binding sites.

Conservation of residues is calculated by using the von-Neumann-Entropy in combination with the substitution matrix BLOSUM62 (details are given in [18]).

We evaluate the descriptors' accuracy in terms of standard precision and recall, where precision is defined as TP/(TP+FP) and recall is defined as TP/(TP+FN). TP, FP, and FN denote the numbers of true positives, false positives and false negatives, respectively.

## List of abbreviations

ATP – Adenosine triphosphate, PLI – Protein-Ligand Interaction, PPI – Protein-Protein Interaction, HMM – Hidden Markov Model, SCOP – Structural Classification Of Proteins, SCOPPI – Structural Classification Of Protein-Protein Interactions, PDB – Protein Databank, HSSP – Homology-derived secondary structure of proteins, MSA – Multiple Sequence Alignment, TP/FP/TN/FN – True/False Positives/Negatives, resp.

## Competing interests

The authors declare that they have no competing interests.

## Authors' contributions

AH: Concept, Implementation, Validation, Manuscript; CW: Validation, Manuscript; WK: Concept; MS: Supervision, Concept

## Supplementary Material

Additional file 1 Individual recall results per face descriptor. Cross validation results for each interface type. Click here for file

## Figures and Tables

**Table 1:** Matches for ATP- and ADP-binding descriptors with various E-value thresholds.

**Hits**	**Precision**	**Recall**	**Threshold**	**TP**	**FP**	**New**
2873	96.59	7.55	1.00E-10	2775	98	771
3113	96.5	8.17	1.00E-09	3004	109	814
3459	96.18	9.05	1.00E-08	3327	132	854
3943	96.25	10.32	1.00E-07	3795	148	899
4488	95.9	11.7	1.00E-06	4304	184	929
5114	93.86	13.05	1.00E-05	4800	314	955
5773	92.74	14.56	0.0001	5354	419	982
6477	91.71	16.15	0.001	5940	537	1012
7477	91.53	18.61	0.01	6844	633	1053
8749	91.45	21.76	0.1	8001	748	1115
**10349**	**89.43**	**25.17**	**1**	**9255**	**1094**	**1249**
14165	78.86	30.38	10	11171	2994	1499
27926	53.67	40.76	100	14988	12938	2440
93261	25.85	65.56	1000	24109	69152	6397

A benchmark set containing 205.000 known ATP-binding or not ATP-binding SwissProt entries was used to measure the precision and recall of 306 ADP/ATP binding descriptors. Hits were counted at various E-value cutoffs. Number of True Positives (TP), False Positives (FP) and number of those True Positives not found automatically by any Prosite-pattern (New) were determined.

**Table 2:** Literature evidence.

Number of interactions found	Percentage	E-value cutoff
230	1.6	0.001
266	1.9	0.01
304	2.1	0.1
348	2.5	1
2.226	15.7	10
11.157	78.8	100

Using 740 face descriptor pairs, a benchmark set of 14,150 high-confidence literature-curated interactions was searched. With a relaxed E-value cutoff we are able to match 78.8% of the literature interactions, thus pinpointing probable interface residues.

## References

[B1] Galperin MY, Koonin EV (2000). Who's your neighbor? New computational approaches for functional genomics. Nat Biotechnol.

[B2] Tong AHY, Lesage G, Bader GD, Ding H, Xu H, Xin X, Young J, Berriz GF, Brost RL, Chang M, Chen Y, Cheng X, Chua G, Friesen H, Goldberg DS, Haynes J, Humphries C, He G, Hussein S, Ke L, Krogan N, Li Z, Levinson JN, Lu H, Menard P, Munyana C, Parsons AB, Ryan O, Tonikian R, Roberts T, Sdicu AM, Shapiro J, Sheikh B, Suter B, Wong SL, Zhang LV, Zhu H, Burd CG, Munro S, Sander C, Rine J, Greenblatt J, Peter M, Bretscher A, Bell G, Roth FP, Brown GW, Andrews B, Bussey H, Boone C (2004). Global mapping of the yeast genetic interaction network. Science.

[B3] Pellegrini M, Marcotte EM, Thompson MJ, Eisenberg D, Yeates TO (1999). Assigning protein functions by comparative genome analysis: protein phylogenetic profiles. Proc Natl Acad Sci USA.

[B4] Sun J, Xu J, Liu Z, Liu Q, Zhao A, Shi T, Li Y (2005). Refined phylogenetic profiles method for predicting protein-protein interactions. Bioinformatics.

[B5] Fraser HB, Hirsh AE, Wall DP, Eisen MB (2004). Coevolution of gene expression among interacting proteins. Proc Natl Acad Sci USA.

[B6] Keskin O, Tsai CJ, Wolfson H, Nussinov R (2004). A new, structurally nonredundant, diverse data set of protein-protein interfaces and its implications. Protein Sci.

[B7] Davis FP, Sali A (2005). PIBASE: a comprehensive database of structurally defined protein interfaces. Bioinformatics.

[B8] Stein A, Russell RB, Aloy P (2005). 3DID: interacting protein domains of known three-dimensional structure. Nucleic Acids Res.

[B9] Aloy P, Russell RB (2006). Structural systems biology: modelling protein interactions. Nature Reviews Molecular Cell Biology.

[B10] Lichtarge O, Sowa ME (2002). Evolutionary predictions of binding surfaces and interactions. Curr Opin Struct Biol.

[B11] Bairoch A (1992). PROSITE: a dictionary of sites and patterns in proteins. Nucleic Acids Res.

[B12] Espadaler J, Romero-Isart O, Jackson RM, Oliva B (2005). Prediction of protein-protein interactions using distant conservation of sequence patterns and structure relationships. Bioinformatics.

[B13] Li H, Li J (2005). Discovery of stable and significant binding motif pairs from PDB complexes and protein interaction datasets. Bioinformatics.

[B14] Bateman A, Haft DH (2002). HMM-based databases in InterPro. Brief Bioinform.

[B15] Eddy SR (1998). Profile hidden Markov models. Bioinformatics.

[B16] Zdobnov EM, Apweiler R (2001). InterProScan-an integration platform for the signature-recognition methods in InterPro. Bioinformatics.

[B17] Bailey TL, Gribskov M (1998). Combining evidence using p-values: application to sequence homology searches. Bioinformatics.

[B18] Caffrey DR, Somaroo S, Hughes JD, Mintseris J, Huang ES (2004). Are protein-protein interfaces more conserved in sequence than the rest of the protein surface?. Protein Sci.

[B19] Kim WK, Ison JC (2005). Survey of the geometric association of domain-domain interfaces. Proteins.

[B20] Kim WK, Henschel A, Winter C, Schroeder M (2006). The Many Faces of Protein-Protein Interactions: A Compendium of Interface Geometry. PLoS Computational Biology.

[B21] Koike A, Takagi T (2004). Prediction of protein-protein interaction sites using support vector machines. Protein Eng Des Sel.

[B22] Bradford JR, Westhead DR (2004). Improved prediction of protein-protein binding sites using a support vector machines approach. Bioinformatics.

[B23] Torrance JW, Bartlett GJ, Porter CT, Thornton JM (2005). Using a Library of Structural Templates to Recognise Catalytic Sites and Explore their Evolution in Homologous Families. J Mol Biol.

[B24] Ofran Y, Rost B (2003). Predicted protein-protein interaction sites from local sequence information. FEBS Lett.

[B25] Obenauer JC, Yaffe MB (2004). Computational prediction of protein-protein interactions. Methods Mol Biol.

[B26] Berman HM, Westbrook J, Feng Z, Gilliland G, Bhat TN, Weissig H, Shindyalov IN, Bourne PE (2000). The Protein Data Bank. Nucleic Acids Res.

[B27] Ashburner M, Ball CA, Blake JA, Botstein D, Butler H, Cherry JM, Davis AP, Dolinski K, Dwight SS, Eppig JT, Harris MA, Hill DP, Issel-Tarver L, Kasarskis A, Lewis S, Matese JC, Richardson JE, Ringwald M, Rubin GM, Sherlock G (2000). Gene ontology: tool for the unification of biology. The Gene Ontology Consortium. Nat Genet.

[B28] Bateman A, Birney E, Durbin R, Eddy SR, Howe KL, Sonnhammer EL (2000). The Pfam protein families database. Nucleic Acids Res.

[B29] Murzin AG, Brenner SE, Hubbard T, Chothia C (1995). SCOP: A Structural Classification of Proteins Database for the Investigation of Sequences and Structures. Journal of Molecular Biology.

[B30] Winter C, Henschel A, Kim WK, Schroeder M (2006). SCOPPI: A Structural Classification of Protein-Protein Interfaces. Nucleic Acids Res.

[B31] Sander C, Schneider R (1991). Database of homology-derived protein structures and the structural meaning of sequence alignment. Proteins.

[B32] Scordis P, Flower DR, Attwood TK (1999). FingerPRINTScan: intelligent searching of the PRINTS motif database. Bioinformatics.

[B33] Grundy WN, Bailey TL, Elkan CP, Baker ME (1997). Meta-MEME: motif-based hidden Markov models of protein families. Comput Appl Biosci.

[B34] http://www.molecularconnections.com.

[B35] Walhout AJ, Sordella R, Lu X, Hartley JL, Temple GF, Brasch MA, Thierry-Mieg N, Vidal M (2000). Protein interaction mapping in C. elegans using proteins involved in vulval development. Science.

[B36] Aloy P, Russell RB (2002). Interrogating protein interaction networks through structural biology. Proc Natl Acad Sci USA.

[B37] Deane CM, Salwinski L, Xenarios I, Eisenberg D (2002). Protein interactions: two methods for assessment of the reliability of high throughput observations. Mol Cell Proteomics.

[B38] Aytuna A, Gursoy A, Keskin O (2005). Prediction of protein-protein interactions by combining structure and sequence conservation in protein interfaces. Bioinformatics.

[B39] Edgar RC (2004). MUSCLE: multiple sequence alignment with high accuracy and high throughput. Nucleic Acids Res.

[B40] Crooks GE, Hon G, Chandonia JM, Brenner SE (2004). WebLogo: a sequence logo generator. Genome Res.

