# Supplementary material for: Using structural motif descriptors for sequence-based binding site prediction
Source: BMC Bioinformatics. 2007 May 22;8(Suppl 4):S5. doi: 10.1186/1471-2105-8-S4-S5 (PMC1892084; doi:10.1186/1471-2105-8-S4-S5)
Supplement: Additional file 1 — Individual recall results per face descriptor. Cross validation results for each interface type. [file 1471-2105-8-S4-S5-S1.doc]

## Additional file 1 – Individual recall results per face descriptor

Cross validation results for each interface type: The first four columns denote the interface type consisting of the two SCOP family ids and the respective face.

| Family 1 | Family 2 | Face 1 | Face 2 | Side | Recall | Entropy | Descr.-Length |
| --- | --- | --- | --- | --- | --- | --- | --- |
| a.2.11.1 | d.44.1.1 | 7 | 3 | Left | 100 | 0.3 | 43 |
| a.2.11.1 | d.44.1.1 | 7 | 3 | Right | 98.83 | 0.29 | 52 |
| a.2.11.1 | d.44.1.1 | 8 | 1 | Left | 94.41 | 0.29 | 19 |
| a.2.11.1 | d.44.1.1 | 8 | 1 | Right | 100 | 0.27 | 16 |
| a.27.1.1 | c.26.1.1 | 3 | 15 | Left | 75 | 0.4 | 39 |
| a.27.1.1 | c.26.1.1 | 3 | 15 | Right | 0 | 0.47 | 25 |
| a.29.3.1 | e.6.1.1 | 7 | 1 | Left | 87.88 | 0.29 | 72 |
| a.29.3.1 | e.6.1.1 | 7 | 1 | Right | 89.74 | 0.29 | 83 |
| a.45.1.1 | c.47.1.5 | 3 | 3 | Right | 100 | 0.46 | 22 |
| a.45.1.1 | c.47.1.5 | 6 | 1 | Right | 100 | 0.48 | 40 |
| a.60.7.1 | c.120.1.2 | 1 | 1 | Left | 100 | 0.27 | 64 |
| a.60.7.1 | c.120.1.2 | 1 | 1 | Right | 100 | 0.26 | 87 |
| a.83.1.1 | d.128.1.2 | 4 | 1 | Left | 100 | 0.19 | 48 |
| a.83.1.1 | d.128.1.2 | 4 | 1 | Right | 100 | 0.16 | 52 |
| b.1.1.2 | d.19.1.1 | 30 | 22 | Left | 0 | 0.13 | 8 |
| b.1.1.2 | d.19.1.1 | 30 | 22 | Right | 0 | 0.19 | 4 |
| b.1.1.2 | d.19.1.1 | 30 | 23 | Left | 0 | 0.19 | 14 |
| b.1.1.2 | d.19.1.1 | 30 | 23 | Right | 40 | 0.26 | 11 |
| b.1.1.2 | d.19.1.1 | 30 | 32 | Left | 29.03 | 0.18 | 18 |
| b.1.1.2 | d.19.1.1 | 30 | 32 | Right | 100 | 0.24 | 19 |
| b.1.1.2 | d.19.1.1 | 30 | 36 | Left | 100 | 0.16 | 13 |
| b.1.1.2 | d.19.1.1 | 30 | 36 | Right | 100 | 0.23 | 14 |
| b.1.1.2 | d.19.1.1 | 31 | 32 | Left | 100 | 0.07 | 23 |
| b.1.1.2 | d.19.1.1 | 31 | 32 | Right | 97.78 | 0.24 | 42 |
| b.1.1.2 | d.19.1.1 | 31 | 33 | Left | 100 | 0.17 | 22 |
| b.1.1.2 | d.19.1.1 | 31 | 33 | Right | 100 | 0.22 | 32 |
| b.19.1.2 | h.3.1.1 | 6 | 1 | Left | 100 | 0.19 | 83 |
| b.19.1.2 | h.3.1.1 | 6 | 1 | Right | 0 | 0.12 | 116 |
| b.34.3.1 | c.37.1.9 | 2 | 8 | Left | 100 | 0.21 | 18 |
| b.34.3.1 | c.37.1.9 | 2 | 8 | Right | 100 | 0.16 | 28 |
| b.35.1.2 | c.2.1.1 | 6 | 5 | Left | 85.91 | 0.34 | 14 |
| b.35.1.2 | c.2.1.1 | 6 | 5 | Right | 0 | 0.35 | 16 |
| b.35.1.2 | c.2.1.1 | 8 | 1 | Left | 0 | 0.41 | 80 |
| b.35.1.2 | c.2.1.1 | 8 | 1 | Right | 63.84 | 0.43 | 65 |
| b.40.2.2 | d.15.6.1 | 6 | 9 | Left | 100 | 0.41 | 61 |
| b.40.2.2 | d.15.6.1 | 6 | 9 | Right | 100 | 0.38 | 63 |
| b.43.3.1 | b.44.1.1 | 4 | 1 | Left | 69.7 | 0.25 | 21 |
| b.43.3.1 | b.44.1.1 | 4 | 1 | Right | 8.89 | 0.3 | 13 |
| b.43.4.2 | c.25.1.1 | 5 | 1 | Left | 78.57 | 0.24 | 44 |
| b.43.4.2 | c.25.1.1 | 5 | 1 | Right | 100 | 0.26 | 50 |
| b.47.1.2 | g.3.2.1 | 18 | 1 | Left | 100 | 0.16 | 47 |
| b.47.1.2 | g.3.2.1 | 18 | 1 | Right | 100 | 0.13 | 20 |
| b.47.1.2 | g.68.1.1 | 18 | 5 | Left | 0 | 0.18 | 40 |
| b.47.1.2 | g.68.1.1 | 18 | 5 | Right | 100 | 0.28 | 26 |
| b.47.1.2 | g.8.1.1 | 18 | 7 | Left | 0 | 0.2 | 37 |
| b.47.1.2 | g.8.1.1 | 18 | 7 | Right | 100 | 0.1 | 19 |
| b.52.2.2 | c.81.1.1 | 3 | 4 | Left | 98.39 | 0.39 | 77 |
| b.52.2.2 | c.81.1.1 | 3 | 4 | Right | 83.12 | 0.39 | 88 |
| b.58.1.1 | c.1.12.1 | 1 | 1 | Left | 100 | 0.2 | 22 |
| b.58.1.1 | c.1.12.1 | 1 | 1 | Right | 90.12 | 0.13 | 31 |
| c.1.14.1 | d.58.9.1 | 9 | 1 | Left | 100 | 0.22 | 49 |
| c.1.14.1 | d.58.9.1 | 9 | 1 | Right | 100 | 0.22 | 54 |
| c.1.8.5 | d.26.3.1 | 11 | 1 | Left | 100 | 0.33 | 54 |
| c.1.8.5 | d.26.3.1 | 11 | 1 | Right | 100 | 0.34 | 48 |
| c.2.1.3 | d.81.1.1 | 25 | 8 | Left | 80.31 | 0.24 | 22 |
| c.2.1.3 | d.81.1.1 | 25 | 8 | Right | 100 | 0.2 | 19 |
| c.2.1.3 | d.81.1.1 | 26 | 3 | Left | 4.07 | 0.25 | 13 |
| c.2.1.3 | d.81.1.1 | 26 | 3 | Right | 100 | 0.2 | 9 |
| c.2.1.3 | d.81.1.1 | 4 | 7 | Left | 0 | 0.43 | 30 |
| c.2.1.3 | d.81.1.1 | 4 | 7 | Right | 52.15 | 0.38 | 46 |
| c.2.1.3 | d.81.1.3 | 5 | 6 | Left | 0 | 0.18 | 33 |
| c.2.1.3 | d.81.1.3 | 5 | 6 | Right | 96.43 | 0.18 | 24 |
| c.2.1.4 | c.23.12.1 | 5 | 1 | Left | 0 | 0.32 | 13 |
| c.2.1.4 | c.23.12.1 | 5 | 1 | Right | 59.09 | 0.36 | 15 |
| c.2.1.5 | d.162.1.1 | 2 | 3 | Left | 86.67 | 0.38 | 29 |
| c.2.1.5 | d.162.1.1 | 2 | 3 | Right | 100 | 0.41 | 45 |
| c.2.1.5 | d.162.1.1 | 3 | 1 | Left | 83.91 | 0.38 | 65 |
| c.2.1.5 | d.162.1.1 | 3 | 1 | Right | 89.15 | 0.39 | 67 |
| c.2.1.7 | c.58.1.1 | 7 | 1 | Left | 100 | 0.31 | 48 |
| c.2.1.7 | c.58.1.1 | 7 | 1 | Right | 94.17 | 0.29 | 43 |
| c.3.1.4 | d.168.1.1 | 8 | 5 | Left | 90 | 0.22 | 78 |
| c.3.1.4 | d.168.1.1 | 8 | 5 | Right | 93.02 | 0.22 | 52 |
| c.3.1.5 | d.87.1.1 | 3 | 3 | Left | 48.65 | 0.4 | 34 |
| c.3.1.5 | d.87.1.1 | 3 | 3 | Right | 84.78 | 0.4 | 32 |
| c.3.1.5 | d.87.1.1 | 5 | 8 | Left | 0 | 0.36 | 41 |
| c.3.1.5 | d.87.1.1 | 5 | 8 | Right | 26.51 | 0.36 | 18 |
| c.3.1.5 | d.87.1.1 | 9 | 6 | Left | 60.34 | 0.38 | 36 |
| c.3.1.5 | d.87.1.1 | 9 | 6 | Right | 85.33 | 0.39 | 24 |
| c.31.1.3 | c.36.1.9 | 10 | 1 | Left | 66.67 | 0.39 | 36 |
| c.31.1.3 | c.36.1.9 | 10 | 1 | Right | 93.44 | 0.36 | 49 |
| c.36.1.5 | c.36.1.9 | 9 | 2 | Left | 90.7 | 0.28 | 38 |
| c.36.1.5 | c.36.1.9 | 9 | 2 | Right | 93.44 | 0.31 | 40 |
| c.55.1.1 | d.109.1.1 | 14 | 8 | Left | 100 | 0.04 | 31 |
| c.55.1.1 | d.109.1.1 | 14 | 8 | Right | 94.12 | 0 | 43 |
| c.55.3.5 | e.8.1.1 | 5 | 7 | Left | 0 | 0.24 | 35 |
| c.55.3.5 | e.8.1.1 | 5 | 7 | Right | 100 | 0.22 | 42 |
| d.175.1.1 | e.3.1.1 | 4 | 3 | Left | 100 | 0.15 | 55 |
| d.175.1.1 | e.3.1.1 | 4 | 3 | Right | 100 | 0.24 | 51 |
